# A Comparative Assessment of Right-Angle Screwdriver Versus Conventional Screwdriver for Orthognathic Surgeries

**DOI:** 10.7759/cureus.58822

**Published:** 2024-04-23

**Authors:** Bharan Ravindran, Shanmugasundaram Somasundaram, Krishnakumar Raja, Venkata Saikrishna Yalagala, John Rozar Raj

**Affiliations:** 1 Department of Oral and Maxillofacial Surgery, Sri Ramaswamy Memorial (SRM) Ramapuram Dental College and Hospital, Chennai, IND

**Keywords:** intraoral, angulation, fixation, mandible, orthognathic

## Abstract

Background

Bilateral orthognathic surgery is one of the unique and challenging orthognathic surgeries that gained popularity after numerous modifications to the techniques. Using linear equipment inside the oral cavity might lead to malalignment or structural weakness due to incorrect placement, making the ramus osteotomy more difficult. By directly positioning the instrument perpendicularly over the mandibular ramus region, a right-angle drill can help promote a more ergonomic approach.

Objectives

The primary objective was to compare the efficacy of a right-angle screwdriver over a conventional screwdriver for orthognathic surgery using clinical parameters. The second objective was to assess the ease of fixation, the ease of osteotomy, and the time taken for the surgery in both groups.

Methods

This prospective observational study, comparing the effectiveness of conventional and right-angle screwdrivers for orthognathic surgery, was carried out on 12 patients. A split-mouth design was chosen. For the 12 patients, a right-angle screwdriver was used to secure the right mandibular plate and screw, while a traditional straight handpiece was used to fix the left plate and screw. Clinical parameters such as the ease of osteotomy, the ease of fixation, the time taken for fixation, and the postoperative angulation of the screw in relation to the bone with cone beam computed tomography were evaluated. The procedure was communicated to the patients, followed by obtaining written informed consent.

Result

Statistical analysis was done using descriptive and inferential statistics using an independent sample t-test and an unpaired t-test. IBM SPSS Statistics for Windows, Version 26.0 (Released 2019; IBM Corp., Armonk, NY, USA), was used. The level of significance was set at a p-value of <0.05. Compared to a conventional screwdriver, the right-angle screwdriver made osteotomies and fixations easier for 12 patients and also required less surgical time. Statistically, it was shown that ease of fixation had a statistically significant difference among right-angled and conventional screwdrivers (p-value < 0.001), whereas time taken had an insignificant difference (p-value 0.13). The angulation of screw fixation with the right angle showed a consistent result of fixation perpendicular to the bone when compared with conventional screwdrivers providing stable fixation, which was statistically significant (p-value < 0.001).

Conclusion

Compared to the conventional screwdriver used in orthognathic procedures, the right-angle screwdriver ensures solid fixation and eliminates the challenges encountered while drilling and screwing posteriorly positioned screws.

## Introduction

Orthognathic surgery is a unique form of facial surgery since it can greatly enhance the patient’s occlusal function and look, which, in turn, can improve the patient’s self-esteem and general well-being. Bilateral sagittal split osteotomy (BSSO), which can be done with or without maxillary surgery, is the most common kind of orthognathic surgery [1] and indicates excess, deficit, or asymmetries in the horizontal mandible. Trauner and Obwegeser [2] are credited with providing the initial description of the technique, which has undergone numerous improvements. The fundamental osteotomy has been defined by these two alterations together, whereby the bone cut on the mandible’s medial aspect finishes at the lingual fossa, while the bone cut on the lateral aspect extends forward along the external oblique ridge [2].

There are numerous techniques for mandibular fixation, such as the combination of intraosseous wiring and intermaxillary fixation (IMF), which has been associated with a notable rate of recurrence and patient discontent. Rigid fixation, a three-point fixation with positioning screws, is an additional mandibular fixation technique. The use of monocortical screws in 2-mm plate fixation is a novel method of rigid fixation. The switch to rigid fixation improved patient comfort, sped up the healing process, and provided more support for the mandible’s stability following BSSO [3].

Among the issues related to screw fixation are early screw loosening, hardware exposition, skeletal instability or early relapses, and infection [4]. Most rigid fixation surgeries that accompany sagittal split ramus osteotomies of the jaw use the transbuccal technique. A cheek skin incision may result in unfavorable aftereffects, like obvious scarring [5].

Various bone screws have had their mechanical characteristics analyzed. It has been determined how well 4-mm self and non-self-tapping screws hold in the bone as well as how much torque they produce when inserted. Preventing screw failures during insertion can be achieved by utilizing a torque-limiting mechanism while inserting screws into tapped holes [6].

Bouwman et al. evaluated the use of transbuccal positioning screws to stabilize bone segments after a BSSO for mandibular advancement. Seven hundred examples in a row were evaluated. In 19 patients (2.7% of instances), there was no utilization of screw fixation as the fixing technique. Twenty patients (2.8% of the total) had their screws removed; 15 of these instances had infections, and five had other reasons. Three of the patients had a reoperation due to screw loosening that happened in the first postoperative week [7].

The purpose of the 90 ° screwdriver is to facilitate a less invasive intraoral approach for screw insertion and drilling into bone, thereby addressing screw fixation failures and preventing scarring from the transbuccal approach. This study aims to assess if employing a right-angle screwdriver instead of a conventional screwdriver can improve clinical outcomes for screw fixation in mandibular BSSOs.

## Materials and methods

This prospective observational study was conducted at the Department of Oral and Maxillofacial Surgery at Sri Ramaswamy Memorial (SRM) Ramapuram Dental College and Hospital in Chennai, India, to compare the efficacy of a right-angle screwdriver to a conventional screwdriver for orthognathic surgery. The Institutional Ethical Committee approved the study (approval number SRMDC/IRB/2021/MDS/NO.405). Twelve patients who were clinically and conclusively diagnosed with dentofacial deformities undergoing orthodontic surgery, aged between 18 and 30 years, formed the inclusion criteria for the study, whereas any patients who were not willing to take part in the study, patients not willing for long-term follow-up, medically compromised, and syndromic patients, as well as being outside the age limits, were systematically and strictly excluded from the study. The selected patients underwent mandibular advancement or setback surgery, either with or without concurrent maxillary surgery and genioplasty. A split-mouth design was chosen, and the dental arch was divided into two groups: the right-side group and the left-side group, with 12 patients each undergoing orthognathic surgery. In the 12 patients, the right-side mandibular plate and screw fixation were performed with a right-angle screwdriver, while the left-side plate and screw fixation were done with a conventional straight handpiece. The procedure to be performed was communicated to the patient, followed by written informed consent. The age, gender, pertinent demographic information, and medical background of every patient were documented.

The primary outcome of our study was to compare the efficacy of a right-angle screwdriver over a conventional screwdriver for orthognathic surgery using clinical parameters. Secondary outcome measures include the ease of osteotomy, the ease of fixation, and the time taken for the surgery in both conventional and right-angle groups.

The trial included patients who met the inclusion criteria and provided consent. Surgery was performed by a single, skilled surgeon using Epker’s procedure for BSSO. The patients were cleaned and laid supine on the operating table under general nasotracheal intubation, their entire face and neck inside the field, wrapped for an intraoral surgical procedure. Inferior alveolar nerve blocks were administered using a short-acting local anesthetic, supplemented with a long-acting anesthetic if necessary. Anatomical landmarks such as the external oblique ridge and anterior border of the ramus were used to determine the intraoral incision site. An incision was made in the mucosa with electrocautery, just above the anterior border of the ramus midway. The incision continued along the second molar below the external oblique ridge and then descended to the distal first molar after moving much farther laterally into the vestibule. To aid with closure, it is recommended to leave a tissue cuff in place medially to the incision. The periosteum, muscle, and submucosa were all cut through using electrocautery. The proximal mandibular body and all of the tissue along its buccal surface were dissected using a periosteal elevator. The mandibular body’s inferior border and the ramus’s posterior border were accessible by dissection. A lower-border retractor was then attached to the inferior border of the mandible, and each anterior ramus attachment was released onto the coronoid notch as a superior attachment.

The medial portion of the ramus was exposed through subperiosteal dissection, extending along the internal oblique ridge below the level of the occlusal plane. Dissection began superiorly and ended just in front of and behind the lingula, proceeding both inferiorly and posteriorly using a blunt elevator for elevation.

Once soft tissue dissection was complete, attention shifted to the osteotomies. To retract and protect the pedicle, a narrow periosteal elevator was positioned along the medial side of the ramus. The incision was placed parallel to the occlusal plane, superior to the lingula, and medial to the ascending ramus. After cutting through the cortical bone, the cancellous bone was reached. It was then extended distally to the first molar by stretching anteriorly along the external oblique ridge. Subsequently, a vertical incision was made from the inferior border to the level of the first molar along the buccal cortex. Each incision was examined to ensure complete reach of the cancellous bone and cortex. Osteotomes completed the cut from anterior to posterior. As the split opened, the location of the inferior alveolar nerve was verified. If trapped on the proximal or lateral section, it was gently released using a blunt periosteal elevator. Attention was given to ensuring that each segment remained independent and that the condylar head remained linked to the proximal segment after completion of the osteotomy.

**Figure 1:**
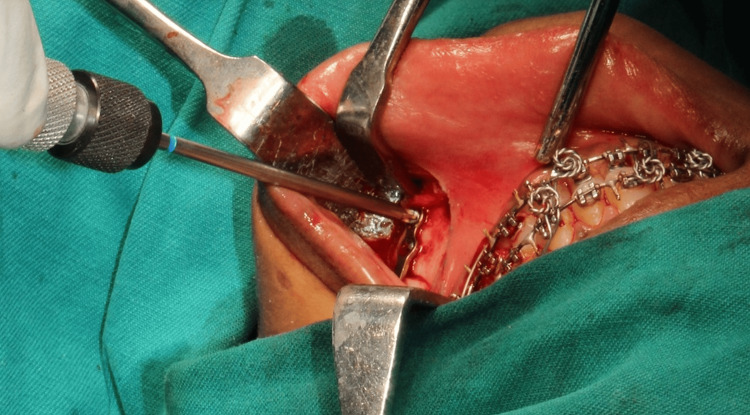
Screw fixation with a conventional screwdriver

**Figure 2:**
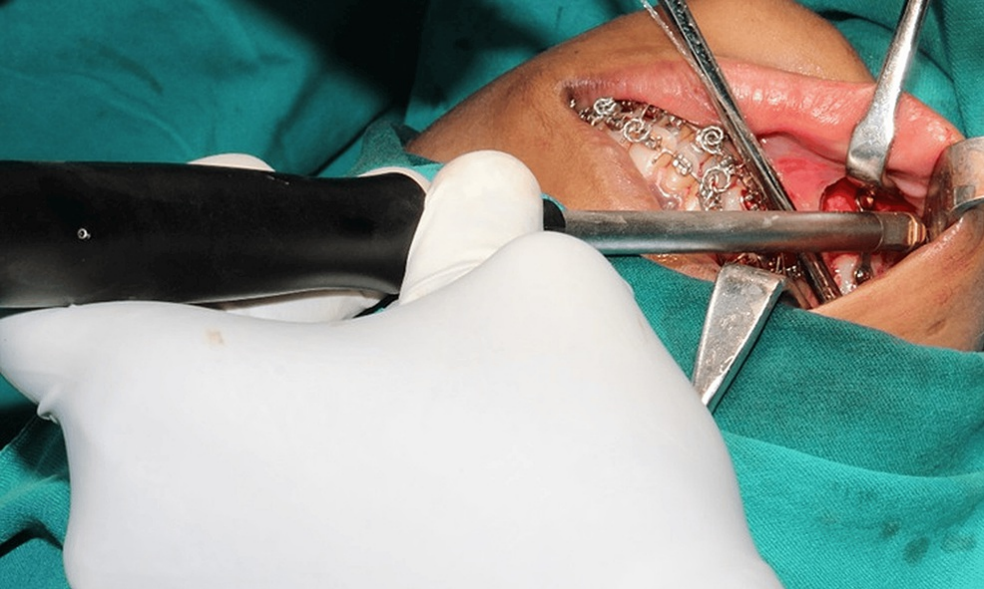
Screw fixation with a right-angle screwdriver

The prefabricated splint was used to adjust the mandible into its correct position. If a mandibular setback was necessary, any intervening bone was removed. The two segments were then joined using a miniplate equipped with four holes and a gap on both sides of the osteotomy. Right-angle handpieces were employed for right-side osteotomies, whereas straight handpieces were used for left-side osteotomies (Figure 1, Figure 2).

A traditional screwdriver was used to fix the screws on the left side, and a right-angle screwdriver was used to secure the titanium screws on the right side. Clinical parameters were assessed and documented, including the ease of osteotomy, the ease of screw fixation, and the fixation time. Fixation was placed with caution to guarantee that the inferior border was properly aligned and that the condyle stayed inside the fossa. After the segments were fixed, occlusion was checked to ensure it was satisfactory. Following extensive irrigation and hemostasis, the incisions were sealed with an absorbable suture if the intended occlusion had been achieved. Guiding elastics could be implanted either during or after surgery.

**Figure 3:**
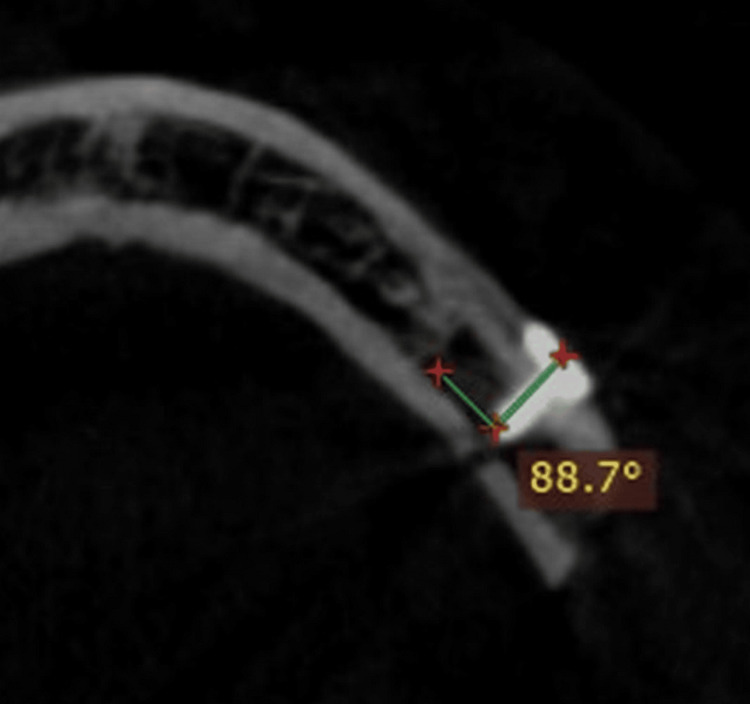
CBCT image showing angulation of screw fixation with a conventional screwdriver CBCT, cone beam computed tomography

**Figure 4:**
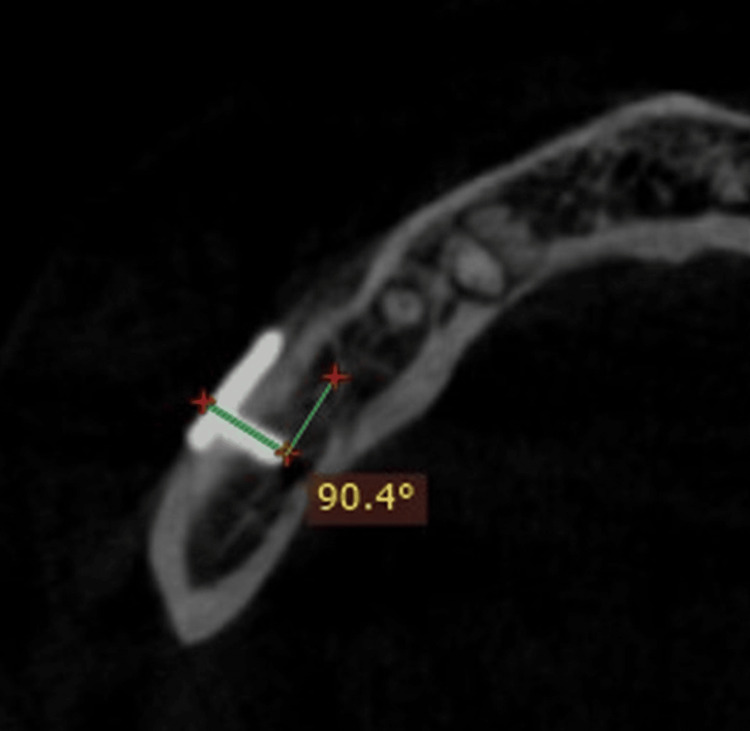
CBCT image showing angulation of screw fixation with a right-angled screwdriver CBCT, cone beam computed tomography

To evaluate the screw’s angulation in relation to the bone, a cone beam computed tomography (CBCT) scan of the facial bones was performed after the seventh postoperative day (Figure 3, Figure 4).

In the normalcy tests based on the results of the Shapiro-Wilk and Kolmogorov-Smirnov tests, values were found to be distributed normally. Thus, the data was analyzed using parametric analysis. In order to express descriptive data, means and standard deviations were used. For inferential statistics, the unpaired t-test and the independent sample t-test were employed. Data analysis was done using IBM SPSS Statistics for Windows, Version 26.0 (Released 2019; IBM Corp., Armonk, NY, USA). There was a predefined significance level of 5% (α = 0.05). p-values below 0.05 were regarded as statistically significant.

## Results

Table 1 shows a comparison of osteotomy techniques among right-angle and conventional screwdrivers. The mean was found to be higher in the conventional screwdriver (3.0) than in the right-angle screwdriver (1.0). Since the individual patients’ values were the same for all the patients, the p-value was not computed.

**Table 1:** Comparison of ease of osteotomy among right-angle and conventional screwdriver

Variables	Right-angle screwdriver	Conventional screwdriver
Mean	1.0000	3.0000
SEM	0.00000	0.00000
SD	0.00000	0.00000
Variance	0.000	0.000
Range	0.00	0.00
t-value	Cannot compute	
p-value		

Table 2 shows the comparison of the ease of fixation between the right-angle and conventional screwdrivers. The mean was found to be higher in the conventional screwdriver (2.41) than in the right-angle screwdriver (1.0). While assessing the statistical difference, it was found that a statistically significant difference was seen between the right-angle and conventional screwdrivers.

**Table 2:** Comparison of ease of fixation among right-angle and conventional screwdriver * indicates p < 0.05

Variables	Right-angle screwdriver	Conventional screwdriver
Mean	1.0000	2.4167
SEM	0.00000	0.14865
SD	0.00000	0.51493
Variance	0.000	0.265
Range	0.00	1.00
t-value	9.53	
p-value	<0.001^*^	

Table 3 shows the comparison of the time taken for fixation between the right-angle and conventional screwdrivers. The mean was found to be higher in the conventional screwdriver (8.26) than in the right-angle screwdriver (7.81). While assessing the statistical difference, it was found that a statistically significant difference was seen between the right-angle and conventional screwdrivers.

**Table 3:** Comparison of time taken for fixation between right-angle and conventional screwdriver

Variables	Right-angle screwdriver	Conventional screwdriver
Mean	7.8183	8.265
SEM	0.30248	0.05166
SD	1.04782	0.17896
Variance	1.098	0.032
Range	2.92	0.6
t-value	1.62	
p-value	0.13	

Table 4 shows the comparison of the angulation of the screw about the bone with CBCT between the right-angle and conventional screwdrivers.

**Table 4:** Comparison of angulation of the screw in relation to the bone with CBCT between the right-angle and conventional screwdriver * indicates p < 0.05 L = left side (conventional drill); R = right side (right-angle drill) CBCT, cone beam computed tomography

Variables	First screw		Second screw		Third screw		Fourth screw	
	R	L	R	L	R	L	R	L
Mean	90.2917	85.7417	90.0750	84.6417	90.0917	85.8000	90.2500	85.2333
SEM	0.08569	0.61490	0.07600	0.49566	0.07330	0.46090	0.10038	0.54430
SD	0.29683	2.13007	0.26328	1.71700	0.25391	1.59659	0.34772	1.88551
Variance	0.088	4.537	0.069	2.948	0.064	2.549	0.121	3.555
Range	0.80	6.30	0.90	5.50	0.90	4.90	1.10	6.60
t-value	7.7		10.2		9.7		8.9	
p-value	<0.001^*^		<0.001^*^		<0.001^*^		<0.001^*^	

While assessing the first screw, the mean was higher for the right-angle screwdriver (90.29) than the conventional screwdriver (85.74). The statistical difference showed that a significant difference was seen between the right-angle and conventional screwdrivers. While assessing the second screw, the mean was higher for the right-angle screwdriver (90.07) than the conventional screwdriver (84.64). The statistical difference showed that a significant difference was seen between the right-angle and conventional screwdrivers. While assessing the third screw, the mean was higher for the right-angle screwdriver (90.09) than the conventional screwdriver (85.80). The statistical difference showed that a significant difference was seen between the right-angle and conventional screwdrivers. While assessing the fourth screw, the mean was higher for the right-angle screwdriver (90.25) than the conventional screwdriver (85.23). The statistical difference showed that a significant difference was seen between the right-angle and conventional screwdrivers.

## Discussion

Patients too elderly for growth modification or with dentofacial abnormalities too severe for orthodontic or surgical camouflage typically have the cornerstone treatment of orthognathic surgery to move the mandible, chin, or maxilla forward or backward. Standard orthognathic operations are used to correct jaw distortion as part of the orthodontic surgical treatment for dentofacial deformity. Various adjuvant techniques are also used to gradually enhance the hard and soft tissue shapes [1].

One of the most common osteotomies used to treat mandibular deformities is likely the BSSO. Stability can be attained in a procedure known as rigid internal fixation (RIF) through the use of contemporary metal plates and screws following osteotomy, without the need for IMF. Because RIF devices, including mini plates and screws, are compliance-independent methods of stabilizing the mandibular segments following BSSO, their development has led to an increase in the application and acceptance of orthognathic surgery. RIF techniques support masticatory function and bone repair following surgery. Moreover, the RIF approach can be used to start the early improvement of oral hygiene [8] instead of intermaxillary rigid fixation.

The main aim of this study was to determine the clinical efficacy of a right-angle screwdriver over a conventional screwdriver for the fixation of screws in the BSSO of the mandible. This split-mouth study comprises 12 patients, with seven males and five females undergoing orthognathic surgery. The BSSO was performed using Epker’s technique. Osteotomy was performed with a right-angled handpiece for the right side and a conventional handpiece for the left side. After the orthognathic surgery, the two bilateral segments were fixed with a four-hole miniplate with a gap using a right-angle screwdriver on the right side and a conventional screwdriver on the left side. Our study recorded clinical parameters such as ease of osteotomy, ease of screw fixation, time taken for fixation, and postoperative CT evaluation of the angulation of the screw about bone on both sides.

A statistically significant difference was noted in the ease of osteotomy between the two groups. Conventional screwdrivers exhibited a higher mean value of 3 compared to right-angle screwdrivers (mean value = 1). The ease of osteotomy for drill holes and fixation is better with the right-angle screwdriver than the conventional one. This outcome was consistent with a 2015 study by Vajgel et al. that used an angulated screwdriver to treat condylar fractures utilizing an intraoral technique [9].

In the present study, ease of fixation was observed for the right-angle screwdriver with a mean value of 1, whereas the conventional side showed a higher mean value of 2.4 (p-value < 0.001*). The surgical time taken for the fixation was longer with conventional screwdrivers, with a mean value of 8.26 min. The applied technique should be less time-consuming, as it enhances precision and produces better results with higher patient acceptance. In our experience, it takes less time to insert screws as the surgeon gains proficiency and skill with this right-angle screwdriver.

We have also evaluated the angulation of four screws placed in relation to the bone with the help of CBCT taken on the seventh postoperative day. Different ranges of angulation of the screw in relation to bone include 45-60 º, 60-90 º, and 90 º. The postoperative angulation was 90.29 ± 0.08: 85.7 ± 0.61 for the first screw; 90.07 ± 0.07 and 84.6 ± 0.49 for the second; 90.09 ± 0.07 and 85.8 ± 0.46 for the third; and 90.25 ± 0.10 and 85.23 ± 0.54 for the fourth screw on the right and left sides, respectively. Statistically significant differences were noted when comparing the angulation of four screws in both groups, with a p-value of <0.001*. Moreover, placement of the posterior distal screw will be oblique and difficult with a conventional screwdriver, whereas the right angle gives perpendicular placement of screws to the bone, providing stable and rigid fixation. The right-angle screwdriver has a steep learning curve compared to the conventional one. A certain pattern of repetition and difficulty determines how knowledge or skills are learned. The patterns in which specific talents are mastered are provided by the learning curve. The right-angle screwdriver used for fixation following orthognathic surgery has a steep learning curve, making it challenging to use at first. However, with practice, operating time can be decreased, and the tool performs similarly to a normal screwdriver [10,11].

The right-angle screwdriver avoids the use of transbuccal approaches with trocar and cannula, thereby avoiding extraoral scars. However, avascular necrosis of the proximal segment, bleeding from the collapse of an internal maxillary artery, scarring of the face, and related morbidity are potential side effects of the transbuccal procedure. Damage to the facial nerve continues to be the primary concern. While long-term consequences range from 0% to just 4%, the reported incidence of facial nerve injury with various extraoral treatments ranges from 0 to 24% [12,13].

The extraoral method selected also affects the occurrence of facial nerve paresis. As a result, the extraoral method has very low morbidity and very good standard results. Even if this is a good thing, the scar and the varying periods when facial nerve paresis happen nonetheless cause psychological stress. Troulis suggested utilizing the transoral approach even for displaced condylar mandibular fractures with medial override after five years of practice [14]. Another investigation by Colletti et al. came to the conclusion that an intraoral method was a viable substitute for transcutaneous submandibular access in the therapy of subcondylar fractures [15]. There is no risk to the marginal mandibular nerve, despite Boehle et al.'s evaluation of endoscopic open reduction and internal rigid fixation of subcondylar fractures, reporting an incidence of 4.5% [16]. In addition, the transoral method requires less intrusive surgery than the extraoral method.

However, a number of complications associated with the transoral approach were reported in Wan et al.'s study. These included the plate being positioned in an anatomically unfavorable manner, thin soft tissue coverage increasing the risk of dehiscence and plate exposure, and plate breaking due to increased intraoperative plate bending, which required adjusting to the intricate contours of the mandible’s superior border. Lower bone density on the superior aspect of the mandible and the alveolus, which increases the frequency of screw loosening, and an easier and faster path for bacterial pathogens to travel from the periodontal sulcus to the fixation hardware are additional drawbacks of placing the plate closer to the dentition [17]. However, our study did not report any such complications with the transoral approach except for screw loosening, which was observed in one case with a conventional screwdriver. The angulation of the distal two screws in the observed case with screw loosening was 87.6 ° and 84.6 °, respectively, with the conventional screwdriver, whereas the right-angle screwdriver showed angulations of 90.2 ° and 89.9 °, respectively, in the two posterior-most positioned screws, which were consistent in angulation and placed perpendicular to the bone, which improved the stability of fixation, thereby preventing the complications associated with screw loosening.

Specialized instruments, including reduction forceps, modified nerve hooks, the angulated screwdriver system, extended periosteal elevators and retractors, and endoscopes, have made the reduction and fixation of fractures much easier. Intraoral techniques for treating fractures are now more feasible, thanks to these tools [9]. Reliable force transfer is made possible by the right-angle screwdriver’s minimal weight and efficient drive design. It can be considered a good option to use a right-angle screwdriver for an intraoral vertical ramus osteotomy. In less invasive maxillofacial surgery, an angulated screwdriver seems to be a significant new tool that is helpful, efficient, and good for the clinical result. The screwdriver is made specifically for predrilling and screw insertion, allowing for minimal intrusive treatment. Because of this, linear access is not necessary, and screws can be positioned perpendicular to the osseous surface. Activation lowers the loading force along the screw and lessens the possibility of inadvertent slippage and damage to surrounding important structures because it occurs at a remote pivot point above the handle rather than in line with the insertion axis [18]. Based on our observation, it is suggested to incorporate a smaller head size of the screwdriver in the instrument design and also to minimize the length of the shank to prevent wobbling of the instrument during osteotomy, thereby improving the overall efficacy in the inaccessible areas, which can further enhance the clinical outcome.

Limitations of the study include the fact that the sample size is small. More cases with longer follow-up times show the stability of the fixation, and thorough clinical studies are advised.

## Conclusions

This procedure has the advantages of easy access to the posterior ramus region, leaving no visible scars. Even though surgical fixation with the right-angle screwdriver has a steep learning curve with experience, surgeons can easily perform with better biomechanical stability. As it makes it easier to put screws in without exerting pressure, we recommend including a right-angled screwdriver in the maxillofacial surgical toolbox.
